# The counterion–retinylidene Schiff base interaction of an invertebrate rhodopsin rearranges upon light activation

**DOI:** 10.1038/s42003-019-0409-3

**Published:** 2019-05-13

**Authors:** Takashi Nagata, Mitsumasa Koyanagi, Hisao Tsukamoto, Eshita Mutt, Gebhard F. X. Schertler, Xavier Deupi, Akihisa Terakita

**Affiliations:** 1 Department of Biology and Geosciences, Graduate School of Science, Osaka City University, Osaka, 558-8585 Japan; 2 The OCU Advanced Research Institute for Natural Science and Technology (OCARINA), Osaka City University, Osaka, 558-8585 Japan; 3 Laboratory of Biomolecular Research, Division of Biology and Chemistry, Paul Scherrer Institute, CH-5232 Villigen PSI, Switzerland; 4 Department of Biology, ETH Zürich, 8093 Zurich, Switzerland; 5 Condensed Matter Theory Group, Laboratory for Scientific Computing and Modelling, Paul Scherrer Institute, CH-5232 Villigen PSI, Switzerland

**Keywords:** G protein-coupled receptors, Molecular biophysics, Molecular biophysics, G protein-coupled receptors

## Abstract

Animals sense light using photosensitive proteins—rhodopsins—containing a chromophore—retinal—that intrinsically absorbs in the ultraviolet. Visible light-sensitivity depends primarily on protonation of the retinylidene Schiff base (SB), which requires a negatively-charged amino acid residue—counterion—for stabilization. Little is known about how the most common counterion among varied rhodopsins, Glu181, functions. Here, we demonstrate that in a spider visual rhodopsin, orthologue of mammal melanopsins relevant to circadian rhythms, the Glu181 counterion functions likely by forming a hydrogen-bonding network, where Ser186 is a key mediator of the Glu181–SB interaction. We also suggest that upon light activation, the Glu181–SB interaction rearranges while Ser186 changes its contribution. This is in contrast to how the counterion of vertebrate visual rhodopsins, Glu113, functions, which forms a salt bridge with the SB. Our results shed light on the molecular mechanisms of visible light-sensitivity relevant to invertebrate vision and vertebrate non-visual photoreception.

## Abstract (web summary)

Takashi Nagata et al. use UV-visible spectroscopic analysis to show that Ser186 increases the retinylidene Schiff base (SB) pKa of a spider rhodopsin in the inactive state but not after light activation. The change in contribution of Ser186 to the SB pKa suggests that the counterion (Glu181)–SB interaction rearranges upon light activation.

## Introduction

Animals sense light across a broad range of wavelengths, from ultraviolet (UV) to far-red, using visual pigment rhodopsins and related photosensitive proteins^1,2^, collectively known as rhodopsins. Most rhodopsins function as light-sensitive G-protein-coupled receptors and consist of a protein moiety (opsin) and a chromophore (retinal). The opsin has a fold consisting of seven transmembrane α-helices, where retinal is covalently attached to a Lys residue (Lys296, numbering according to the bovine rhodopsin sequence) through a Schiff base (SB) bond in the seventh helix. Although retinal itself is maximally sensitive to UV light, the specific interactions between the chromophore and the surrounding amino acid residues in the binding site enable a wide variety of wavelength sensitivities in rhodopsins^1,3^.

One of the major mechanisms to achieve sensitivity to visible light (i.e., to red-shift the wavelength of maximum absorption (*λ*max) of the chromophore from the UV to the visible region) is the protonation of the nitrogen of the SB linkage between retinal and Lys296^4^. The protonated state of the SB is, however, not stable in a hydrophobic environment such as the transmembrane core of rhodopsins. In most rhodopsins, a negatively charged amino acid residue near the chromophore—termed counterion—stabilizes the proton on the SB by neutralization of the positive charge, resulting in a shift of the pKa of the SB from neutral to alkaline. Therefore, the presence of a counterion is required for visible light absorption^4^. The counterion in bovine rhodopsin has been identified as Glu113^5–7^, which forms a direct salt bridge with the protonated SB (PSB)^8^ and is responsible for its high pKa and involved in its molecular properties^9–13^. Interestingly, although Glu113 is highly conserved within the vertebrate visual rhodopsins including rod and cone visual pigments, most other rhodopsins have a non-charged amino acid residue such as Tyr and Met at position 113^1,14^ (Supplementary Fig. 1a). Rhodopsins can be classified into several groups: Gt (transducin)-coupled rhodopsins, including bovine rhodopsin and other vertebrate visual rhodopsins; Gq-coupled rhodopsins, containing squid rhodopsin, insect visual rhodopsins and melanopsins; Go-coupled rhodopsins; Gs-coupled rhodopsins; Opn3; Opn5; retinochrome/RGR; and peropsin^1,3^. We have previously revealed that the E181Q mutation on squid retinochrome, amphioxus Go-coupled rhodopsin, and amphioxus peropsin causes a dramatic decrease in the pKa of the SB, clearly showing that Glu181 serves as their SB counterion^14,15^. Glu181, which is located in the second extracellular loop connecting transmembrane helices IV and V, is highly conserved throughout rhodopsins^1,14^ (Supplementary Fig. 1a), strongly suggesting that the role of Glu181 is widely common, probably constituting the ancestral counterion. Very recently, we have discovered that Glu94 acts as the counterion in a Gs-coupled cnidarian opsin^16^.

Interestingly, of the three types of counterion in dark-state rhodopsins (at positions 181, 113, and 94), Glu181 has been shown to also play this role in the light-activated (and G protein-activating) photoproduct of bistable rhodopsins. These rhodopsins exhibit a photo-interconvertible reaction between the dark state and its photoproduct; that is, bistable rhodopsins photo-convert to thermally stable photoproducts with a PSB that can be reverted to the original dark state upon absorption of another photon. Specifically, we have previously discovered that Glu181 functions as counterion in the photoproducts of amphioxus Go-coupled rhodopsin and lamprey parapinopsin^14^. Many Gq-, Gt-, and Go-coupled rhodopsins, Opn3, Opn5, and peropsins contain members having bistable nature^3^; thus, it has been suggested that in most bistable rhodopsins, Glu181 serves indeed as a counterion in both the dark state and photoproduct.

Among the diverse bistable rhodopsins, Gq-coupled rhodopsins have been the best studied group as it consists of not only diverse protostome rhodopsins –including visual pigments of molluscs and arthropods– but also deuterostome non-visual rhodopsins, termed melanopsins or Opn4, which are involved in non-visual photoreception processes such as photo-entrainment of circadian rhythms in mammals. Glu181 is conserved in both protostome visual rhodopsins and deuterostome Opn4^14^ (Supplementary Fig. 1a) and is therefore a strong candidate for being their SB counterion; however, it has been questioned whether this residue actually performs this role by in vivo experiments using transgenic flies^17,18^. It is therefore important to determine whether Glu181 serves as a counterion in a Gq-coupled opsin. In addition, the crystal structure of squid rhodopsin, which is the only reported three-dimensional structure of bistable rhodopsin, revealed that the Glu181 side chain is located too far from the SB (>5 Å) to have a direct interaction^19,20^ (Supplementary Fig. 1b). If this residue does perform the role of counterion, there is little information about how it could interact with the PSB in both dark and light-activated states, taking into account the putative conformational differences between the two states. While these questions could have been addressed by site-directed mutagenesis in squid rhodopsin, to our knowledge no study has reported such data.

Previously, we identified a Gq-coupled rhodopsin from the jumping spider *Hasarius adansoni*–spider Rh1^21,22^. Recombinant spider Rh1 expressed in mammalian cultured cells forms a functional photopigment with its *λ*max in the visible region located at 535 nm. In this study, we performed UV-visible spectroscopic analysis of site-directed mutants of spider Rh1 in order to investigate which amino acids contribute to stabilization of the PSB. The E181Q mutant exhibited a dramatic decrease in the SB pKa both in the dark state and the photoproduct, confirming that Glu181 serves indeed as the SB counterion. In addition, substitutions of Ser186 substantially also affected the SB pKa in the dark state but, remarkably, not in the photoproduct. These results suggest that potential hydrogen-bonding interactions involving Glu181 and Ser186 stabilize the PSB in the dark state, and that these interactions may be rearranged during the formation of the photoproduct, resulting in the loss of the stabilizing contribution from Ser186 to the counterion-PSB system.

## Results

### Glu181 serves as a counterion in spider Rh1

**Fig. 1:**
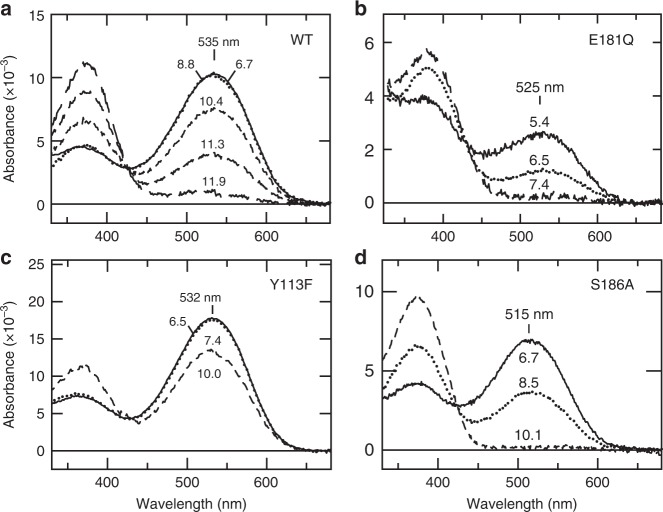
Spectroscopic analysis of the protonation state of the Schiff base in spider Rh1 and its mutants. **a** Absorption spectra of WT spider Rh1 (*λ*max ≈ 535 nm) from neutral (6.7) to alkaline (11.9) pH. **b** Absorption spectra at different pH of the E181Q mutant (*λ*max ≈ 525 nm). **c** Absorption spectra at different pH of the Y113F mutant (*λ*max ≈ 532 nm). **d** Absorption spectra at different pH of the S186A mutant (*λ*max ≈ 515 nm). The pH values at which the spectra were measured are indicated next to the corresponding curves

Mutation of a negatively charged amino acid residue that serves as a counterion results in a marked decrease in the pKa of the SB, thereby resulting in a decrease of protonated SB (with its *λ*max in the visible region) and an increase in the deprotonated form (with *λ*max ≈ 380 nm)^5–7,14,15^. To investigate effects of Glu181 on the PSB in spider Rh1, we examined single amino acid mutants in which Glu181 was substituted with Gln (E181Q) or Asp (E181D). The purified pigment of the wild type (WT) rhodopsin had a main peak at 535 nm and exhibited no pH-dependent change in the absorption spectrum between pH 6.7 and 8.8 (Fig. 1a). In contrast, the E181Q mutant exhibited pH dependence and had almost no peak in the visible region at pH 7.4, indicating a considerable decrease of the SB pKa (Fig. 1b). This result demonstrates that Glu181 serves as the SB counterion in spider Rh1. On the other hand, E181D substitution, which did not result in a large decrease in the SB pKa, resulted in an 8-nm blue shift of *λ*max compared to WT (Supplementary Fig. 2). This is contrary to the effect of the equivalent E113D substitution in the counterion of bovine rhodopsin. It has been reported that the E113D mutant of bovine rhodopsin exhibits a red shift of ~10 nm in its *λ*max, which was explained by weakening of the direct interaction (i.e., salt bridge) between the PSB and the counterion because of the shorter side chain^23^. These results suggest that Glu181 in spider Rh1 interacts with the PSB in a different way from that in bovine rhodopsin, probably involving an indirect and yet unknown interaction(s) mediated by hydrogen bonds.

### Ser186 contributes to stabilization of the PSB

Spider Rh1 and squid rhodopsin share a 35% sequence identity, suggesting a relatively high structural similarity^24^. This allows using the crystal structure of squid rhodopsin (PDBid: 2Z73) as a guide to analyze the counterion-PSB system in spider Rh1 (Supplementary Fig. 1b). Inspection of the structure of squid rhodopsin reveals several amino acid residues within hydrogen-bonding distance from the SB (Asn90, Tyr113, and Asn186) that could form the hypothetic hydrogen-bonding interaction with the SB (Supplementary Fig. 1b). In spider Rh1, position 90 holds a hydrophobic Met; therefore, we investigated whether Tyr113 and Ser186 are involved in stabilization of the PSB. While we did not observe any obvious decrease in the SB pKa in the Y113F mutant, compared to WT, the S186A mutant exhibited a substantial decrease in the SB pKa (Fig. 1c, d). These results show that Ser186 participates in stabilization of the PSB.

**Fig. 2:**
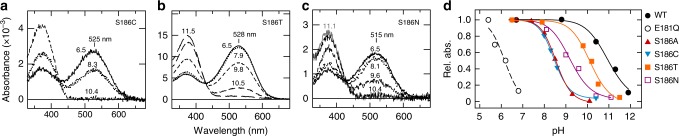
Substitution of Ser186 affects the SB pKa of the dark state. **a**–**c** Absorption spectra of the S186C, S186T, and S186N mutants at different pH values. **d** Relationship between absorbance at *λ*max and pH for the Ser186 mutants. The SB pKa values estimated from the fitting curves are 11.0 (WT), 6.1 (E181Q), 8.6 (S186A), 8.5 (S186C), 10.1 (S186T), and 9.2 (S186N). Numbers on the spectra indicate pH values at which the spectra were measured

To further investigate the role of Ser186, we substituted this residue with other small and neutral amino acids, i.e., Cys, Thr, and Asn (Fig. 2a–c). The S186C mutant exhibited a substantial decrease in the SB pKa to a value of 8.5, similar to the S186A mutant, which is an intermediate value between those of WT and the E181Q mutant (Fig. 2d). On the other hand, the S186T and S186N mutants which retain the polar character of this position exhibited higher pKa values (10.1 and 9.2, respectively) than the S186A and S186C mutants. As this site is close to both the SB and Glu181 (according to the proposed structural homology between squid rhodopsin and spider Rh1; Supplementary Fig. 1b) our data suggest that Ser186 in spider Rh1 could contribute to the stabilization of the PSB in the dark state by forming hydrogen bond interactions with the PSB and/or Glu181.

### Stabilization of the PSB in the photoproduct

**Fig. 3:**
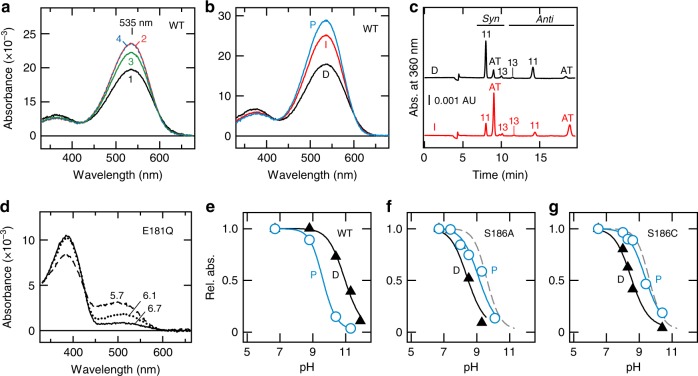
Substitution of Glu181, but not Ser186, affects the SB pKa of the stable photoproduct. **a** Spectral changes in wild type spider rhodopsin upon sequential illumination. The purified pigment was illuminated at pH 6.5 with red (>610 nm), green (≈500 nm), and red (>610 nm) light in this order. The absorption spectra were measured in the darkness (1) and after each illumination (2–4). **b**, **c** Absorption spectra (**b**) and HPLC profile (**c**) of dark (D, black) and illuminated (I, red) pigment (>610 nm). The spectrum of photoproduct (P, blue) in **b** was calculated using the relative amounts of 11-*cis* (11) isomers (dark; **c**, black trace) and both 11-*cis* and all-*trans* (AT) isomers (illuminated; **c**, red trace). In these calculations, we did not consider contamination by all-*trans* and 13-*cis* (13) retinal found in the dark state (**c**, black trace; approximately 17% of all isomers) (see Methods). **d** Calculated absorption spectra of the E181Q photoproduct at different pH values. **e**–**g** Relative absorbance values of WT (**e**), S186A (**f**), and S186C (**g**) mutants in their photoproduct (P, blue) and the dark state (D, black) at *λ*max at different pH values (Fig. 2 shows the corresponding data for the dark states). Dashed lines represent the fitted curve for WT photoproduct

We next asked whether Glu181 and Ser186 play the same roles in the photoproduct as in the dark state. Illumination of spider Rh1 with red light (<610 nm) at a neutral pH caused an increase in absorbance around 535 nm (curves 1 and 2 in Fig. 3a), showing the formation of a stable photoproduct with a PSB. This absorbance around 535 nm decreased upon the second illumination with blue green light (≈500 nm, curve 3) and reverted to the same level as curve 2 upon the third illumination with the same red light (curve 4), proving that spider Rh1 is a bistable rhodopsin and forms different photo-equilibria between the dark state and the stable photoproduct under red and blue green lights. To estimate a pure spectrum of the photoproduct, we measured spectra of samples before and after illumination (Fig. 3b) and then analyzed the configurations of the chromophore retinal in those samples by high-performance liquid chromatography (Fig. 3c) to obtain ratios of 11-*cis* and all-*trans* forms. With these ratios, we calculated the spectrum by assuming that all the original pigment bearing 11-*cis*-retinal (dark state, D in Fig. 3b) was photo-converted to a pigment bearing all-*trans*-retinal (photoproduct, P in Fig. 3b). The calculated spectrum shows that the photoproduct has its *λ*max at around 535 nm and a higher absorption coefficient at this wavelength than the dark state. We then calculated spectra of the photoproducts of WT and the E181Q, S186A, and S186C mutants at different pH values. For the E181Q mutant, the protonated form of the photoproduct exhibited pH-dependency around pH 6 (Fig. 3d), dramatically lower than WT (9.7, Fig. 3e), indicating that Glu181 acts as a counterion also in the photoproduct of spider Rh1.

For the S186A and S186C mutants, irradiation resulted in formation of photoproducts with a PSB (Supplementary Fig. 3) and caused an increase in absorbance in the visible region even at alkaline pH (conditions in which nearly all the dark state pigments had instead a deprotonated SB). These data show that the SB pKa values in the photoproducts of these mutants are higher than in the dark states (9.1 for S186A and 9.4 for S186C, Fig. 3f, g) and similar to that of WT photoproduct (9.7, Fig. 3e). These results demonstrate that the S186A and S186C substitutions have much smaller effects on the SB pKa of their photoproducts compared to the dark states. Consistent to such smaller effects on the SB pKa, S186A and S186C mutation caused smaller shifts of the *λ*max in the photoproducts compared to those in the dark states (Supplementary Table).

**Fig. 4:**
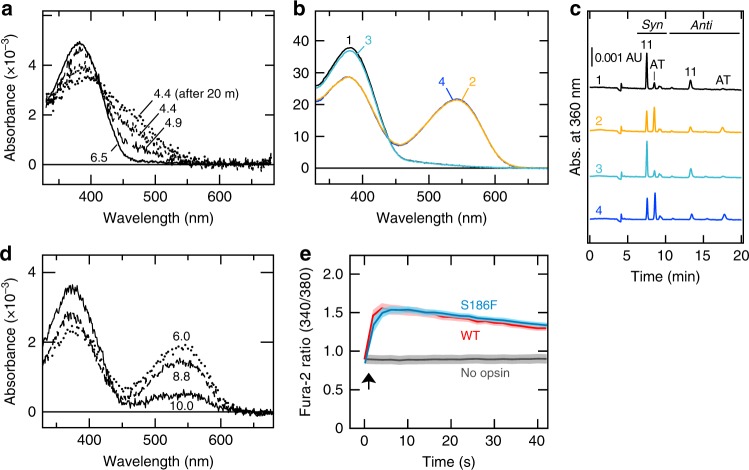
S186F substitution had little effect on the photoproduct. **a** Absorption spectra of the S186F mutant at different pH values. **b**, **c** Changes in the absorption spectra (**b**) and chromophore configurations (**c**) of the S186F mutant after sequential illumination. The S186F mutant was illuminated with violet (≈420 nm), green (≈550 nm), and violet (≈420 nm) light in this order. The absorption spectra were measured in the dark (1) and after each irradiation (2–4). Based on the HPLC profile, approximately 40% of the original 11-*cis*-retinal-bearing pigment was converted to the photoproduct after the first illumination with violet light. **d** Absorption spectra of the S186F mutant photoproduct at different pH values. The S186F mutant photoproduct was produced by irradiation of the purified pigment with violet light (~400 nm) at pH 6.5 and the absorption spectra were measured at pH 6.0, 8.8, and 10.0. **e** Intracellular calcium increase in COS-1 cells expressing WT or the S186F mutant. Cells were stimulated with blue (450–490 nm; WT) or UV (≈380 nm; S186F, and no opsin) light for 1 s between the first and second data points (arrow). Note that similar response amplitudes for WT and the S186F mutant do not necessarily mean similar levels of G protein activation since the cellular responses to light were saturated in the experimental condition. The shaded areas denote the standard error bars calculated on the basis of 15 cells

To further characterize the role of Ser186 in the photoproduct, we continued our analysis of this position by mutagenesis. We observed that the S186F mutation had an extreme effect in the dark state, shifting its *λ*max to the UV region; we did not observe any peak for the protonated form at pH 6.5 (Fig. 4a). Although absorption around 460 nm increased at acidic pH, this component was also increased in a time-dependent manner at pH 4.4, suggesting that this increase reflects the formation of an acid-denatured form. These results suggest that the S186F mutation, unlike S186A and S186C, caused a substantial structural change around the SB. Interestingly, however, irradiation of the dark state (curve 1, Fig. 4b) with violet light resulted in conversion of ~40% of the pigment into the photoproduct (Fig. 4c) with *λ*max at around 540 nm (curve 2, Fig. 4b), similar to the WT (535 nm). This photoproduct reverted to the dark state upon irradiation of green light (≈550 nm; curve 3, Fig. 4b), and such a reversible photoreaction was repeated, showing that the S186F mutant retains a bistable nature. By changing pH after irradiation, the SB pKa of the S186F photoproduct was estimated to be 9–10 (Fig. 4d), which is similar to that of WT. Calcium imaging using the fluorescent calcium indicator fura-2 revealed that cultured cells expressing WT and the S186F mutant exhibited increases in the intracellular calcium level in a light dependent manner (Fig. 4e), demonstrating that the photoproduct of S186F mutant has the ability to activate Gq proteins. In summary, the S186F substitution did not seriously impair the properties of the photoproduct, such as its *λ*max, the SB pKa, or its ability to activate Gq protein. This finding strongly suggests that, in the photoproduct, Ser186 is no longer involved in the Glu181–PSB interaction.

### Effect of T186V mutation in the Go-coupled rhodopsin group

**Fig. 5:**
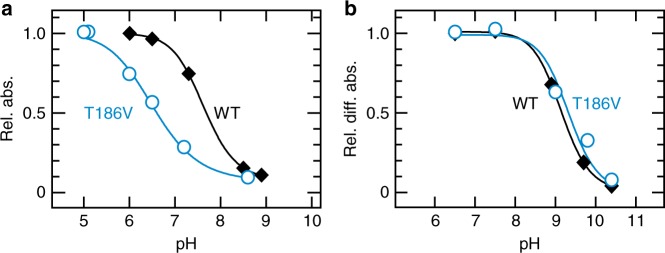
The T186V substitution in amphioxus Go-rhodopsin affects the SB pKa in the dark state but not in the photoproduct. **a** Relative values of absorbance at *λ*max of the dark states of WT (at 485 nm, closed diamonds) and the T186V mutant (at 495 nm, open circles) at different pH values. The estimated pKa values are approximately 7.6 for WT and 6.5 for T186V. **b** Relative values of absorbance at *λ*max of the photoproduct of WT (at 590 nm, closed diamonds) and the T186V mutant (open circles) at different pH values. The estimated pKa values are ~9.1 for WT and 9.3 for T186V

Finally, we aimed to investigate if the residue at position 186 also participates in the counterion-PSB system in other opsin groups with a Glu181 counterion. For this, we mutated position 186 to valine in amphioxus Go-rhodopsin—which has the Glu181 counterion and Thr at position 186 (Supplementary Fig. 1a)—and investigated whether the isosteric T186V substitution affects the pKa of the SB. Compared to WT, the T186V mutant of amphioxus Go-rhodopsin showed a marked decrease in the SB pKa of the dark state (Fig. 5a, Supplementary Fig. 4a, b). The SB pKa values of the photoproducts were estimated in a membrane preparation according to our previous study^14^ (Fig. 5b, Supplementary Fig. 4c, d). The data in Fig. 5b clearly demonstrates that WT and the T186V mutant have a similar SB pKa in the photoproduct. Collectively, our results indicate that the T186V substitution affected the stability of the SB protonation of amphioxus Go-rhodopsin in the dark state but not in the photoproduct, similarly to Ser186 in spider Rh1.

## Discussion

In this study, we have shown that the E181Q substitution results in a substantial decrease of the SB pKa in both the dark state and the photoproduct of spider Rh1. To our knowledge, this is the first biochemical evidence showing that Glu181 stabilizes the protonation of the SB—serving as its counterion—in the Gq-coupled rhodopsin group. We also revealed that Ser186 is involved in the stabilization of the counterion-PSB system in the dark state. In the case of the S186A mutant, a few percent of the pigment is present as the form having the deprotonated SB (Fig. 2d), which is maximally sensitive to UV, at physiological pH (~7.8 ^25^). Such formation of the UV-sensitive form of rhodopsin should negatively affect visual functions of spiders. For instance, sensitivity to color difference (e.g., green vs UV) should decrease because the UV-sensitive form causes UV-sensitivity in the green-sensitive photoreceptor cells in spiders. In addition, the UV-sensitivity in green-sensitive photoreceptor cells should also cause blurs in visual images because UV is extremely blurred in the green-sensitive photoreceptor cell layers due to chromatic aberration of the lens^26^. Such blurs decrease the spatial resolution and, in particular, negatively affect depth perception because the spider utilizes image defocus to perceive depths^22^. Therefore, the contribution of Ser186 to the SB pKa could be physiologically relevant. In the photoproduct, however, substitution of Ser186 had little effect on the SB pKa, demonstrating that the interactions within the counterion-PSB system of the photoproduct are different from that in the dark state. Interestingly, the *λ*max of the photoproduct was not largely affected by substitution of Ser186, unlike the dark state, suggesting different mechanisms for spectral tuning achieving quite similar *λ*max values in the dark state and the photoproduct. Similarly, the T186V mutation on amphioxus Go-rhodopsin lowered the SB pKa in the dark state but not in the photoproduct. These results reveal the possibility that a polar amino acid residue at position 186 is a key mediator of the counterion-PSB system in the dark state of rhodopsins having the Glu181 counterion.

**Fig. 6:**
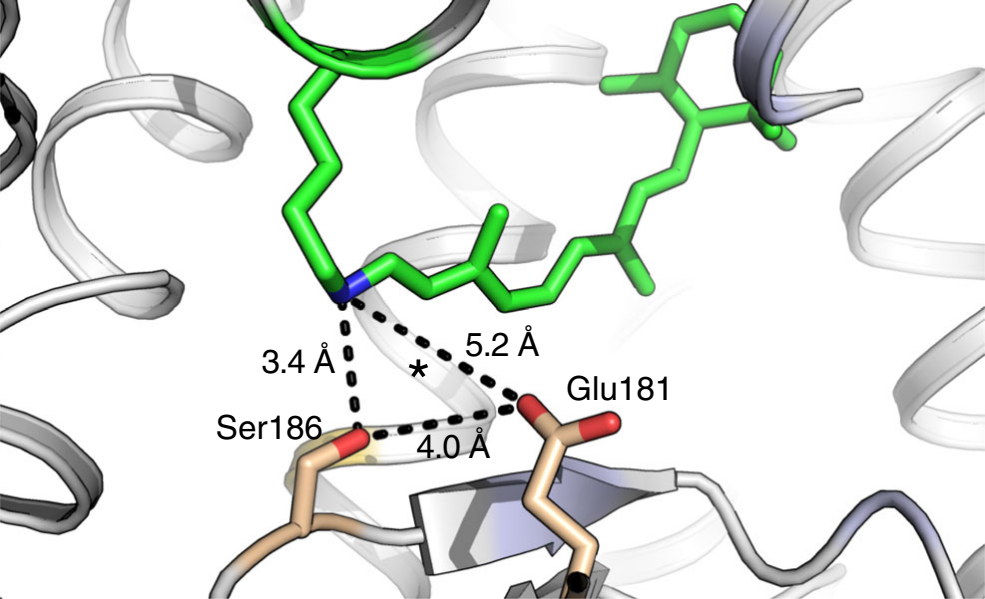
Detail of the counterion-PSB system in our homology model of the dark state of spider Rh1. Distances between Ser186, Glu181, and the PSB are indicated as dashes. It is likely that a water molecule around the position indicated by an asterisk bridging the counterion and PSB extends hydrogen-bonding interactions involving Glu181 and Ser186 to the PSB according to previous reports^19,20,27–30^. See text for details

Our results demonstrate that Glu181 plays the primary role in the stabilization of the PSB in the dark state and the photoproduct of spider Rh1. To obtain clues about how Glu181 stabilizes the PSB, we performed homology modeling to predict the three-dimensional structure of this pigment in the dark state (Fig. 6). In our model, Glu181 is located at a distal position from the SB (>5 Å, as in squid rhodopsin), suggesting that Glu181 could not directly interact with the PSB. However, Ser186 is at a distance of only 3.4 Å from the PSB and 4.0 Å from Glu181. The close proximity of Ser186 to both Glu181 and the PSB suggests that this polar residue could directly or indirectly interact with them via hydrogen-bonding interactions. This hypothesis is consistent with the lower pKa values of the S186A and S186C mutants, as these substitutions eliminate the polar nature of this site and likely destabilize the hydrogen-bonding interactions involving the PSB in the dark state. On the other hand, the S186A and S186C mutants exhibit substantially higher pKa values than the E181Q mutant, suggesting that the PSB–counterion interaction still exists without the OH group of Ser186 side chain. It is possible that the hydrogen-bonding interactions involving Glu181 and Ser186 extend to the PSB via a water molecule (a bridge water). For instance, Ota et al. revealed by FTIR analysis that squid rhodopsin has a strongly hydrogen-bonded water molecule near the SB and proposed it bridges the PSB and Glu181^27^. It has also been known that such a water molecule functions to bridge the counterions and the PSB via hydrogen bonds in the retinal-binding proton pump bacteriorhodopsin^28^. Although X-ray crystallographic studies on squid rhodopsin did not resolve any internal water molecule around the PSB^19,20^, theoretical studies suggest that the space near the PSB observed in the crystal structure might be occupied by water molecules^29,30^. In addition, the crystal structure of bovine rhodopsin^31^ reveals a water molecule hydrogen bonded with Glu181 and Ser186. Based on these data, we propose that in the dark state of spider Rh1, the hydrogen-bonding interactions involving Glu181 and Ser186 extend to the PSB through a water molecule bridging the counterion and the PSB.

The observed effects of the Ser186 mutations are consistent with our bridge water model. Ser186 substitution with non-polar amino acids similar to Ser in volume (i.e., Ala and Cys) resulted in a considerable decrease in the SB pKa. In contrast, substitution for the polar Thr and Asn had milder effects. These data suggest that the side chain of Ser186 could form a hydrogen bond(s) that contributes to the high pKa value of the SB. Although squid rhodopsin with Asn at this position exhibits the SB pKa around 10^32^, the S186N mutant of spider Rh1 exhibited a relatively low pKa value, suggesting that squid rhodopsin may have additional sequence or structure features around this site that assist in raising the SB pKa. The observed complete loss of the SB protonation in the S186F mutant may be partly due to the displacement of the water molecule by the bulky aromatic side chain, which could then impair the PSB–Glu181 interaction. As position 186 holds a Ser/Thr in members of various rhodopsin groups (including melanopsin), direct structural evidence of the hydrogen-bonding network around the counterion-PSB system of a rhodopsin having both Glu181 and Ser186/Thr186—such as spider Rh1—would be invaluable to support the role of this residue and elucidate the full picture of the counterion-PSB system including additional residues other than Glu181 and Ser186.

Our results demonstrate that Glu181 also serves as a counterion in the photoproduct, although in this state Ser186 may not be involved in the stabilization of the PSB. This observation strongly suggests that relative positions of Glu181, Ser186, and the PSB and the hydrogen-bonding interactions connecting them are rearranged during photo-activation. Such a rearrangement is found in bovine rhodopsin, whose crystal structures of the dark and illuminated (Meta II) states are available. Glu181 is 7.1 Å from the PSB in the dark state of bovine rhodopsin but is suggested to serve as the predominant counterion in the Meta I state, which is an inactive photointermediate with its *λ*max in the visible region^33,34^. This indicates that Glu181 could become closer to the PSB to act as a counterion during formation of the Meta I state. Consistently, Glu181 is relatively close to the deprotonated SB (5.0 Å) in the crystal structure of the Meta II state^35^, compared to that in the dark state. In addition, Ser186 is slightly more distant from the SB in the Meta II state than in the dark state (Supplementary Fig. 5). A similar conformational change may occur in spider Rh1; that is, Glu181 and Ser186 become closer to and more distant from the PSB, respectively, which may allow Glu181 and the PSB to interact with each other without involvement of Ser186. We cannot exclude the possibility that Ser186 is still involved in the Glu181–PSB interaction in the photoproduct but the effect of loss of the hydrogen-bonding interaction(s) of Ser186 is compensated by an unknown effect in the Ser186 mutants. In this case, a large conformational rearrangement around Ser186 may occur upon photo-activation since the effects of the S186F substitution are completely different between the dark state and the photoproduct. In any case, such rearrangement of hydrogen-bonding interactions may be a mechanism that enables Glu181 to function as a counterion in both the dark (inactive) and photoproduct (active) states. By contrast, in bovine rhodopsin, the SB becomes deprotonated and Glu181 loses its role as a counterion during Meta I-to-Meta II transition. Such formation of the deprotonated active state, which is characteristic of vertebrate visual rhodopsins, requires the specific hydrogen-bonding network around the Schiff base involving Glu113^14,36^. Therefore, the difference in the hydrogen-bonding network between spider Rh1 and bovine rhodopsin may cause the functional difference of Glu181 in the active state of those opsins.

In addition to the contribution of Ser186 to the function of the Glu181 counterion, such functional coupling mediated by a polar residue at this site seems common among many rhodopsins regardless of the position of their counterion. Recently, we have revealed a new counterion position 94 in a Gs-coupled jellyfish opsin—and possibly in other cnidarian opsins—where Arg186 inhibits Glu181 from acting as a counterion likely by neutralizing the charge^16^. In this lineage, therefore, residues 186 and 181 still interact with each other—as we propose for spider Rh1—and may have an unknown function(s) other than stabilization of the PSB. In vertebrate visual pigments, Ser186 is highly conserved and interacts indirectly with Glu181 through a hydrogen-bonding network that affects many aspects of their molecular properties^13,37,38^. In these rhodopsins, a polar residue at position 186 seems to have functions other than stabilization of the PSB, indicating that the residue 186 can have diverse functions based on its close proximity to both the PSB and Glu181. Therefore, Ser186 in rhodopsins having Glu181 counterion may also have functions in addition to stabilization of the PSB. Clues to such unknown functions of Ser186 might be found in UV-sensitive Gq-coupled opsins (e.g., fruit fly rhodopsin 3 and 4^39^, honeybee UV opsin^5,40^, and jumping spider Rh3^22^), where Glu181 and Ser186 are conserved although they presumably have the deprotonated Schiff base in the dark state. In addition, the functional relevance of Ser186 in the photoproduct is totally unknown. It is of interest to know whether and how Ser186 is involved in the difference in the amplitude of the light-induced conformational change between bovine rhodopsin and rhodopsins having Glu181 counterion^12,14^, which may be related to the difference in the counterion position.

## Methods

### Preparation of mutant opsins

Spider Rh1 and amphioxus Go-rhodopsin tagged with the monoclonal antibody Rho 1D4^41^ epitope-sequence were used^14,22,42^. Single point mutations were introduced by using QuikChange Site-Directed Mutagenesis Kit (Stratagene), according to the manufacturer’s instruction.

### Protein expression and sample preparation for spectroscopy

Opsins were expressed in HEK293S cells (a suspension-adapted variant of HEK293 (ATCC, CRL 1573) established by Nathans et al.^43^) and the reconstituted pigments were purified as described elsewhere^40^. In brief, HEK293S cells were transfected with a plasmid DNA by the calcium-phosphate method. The expressed protein was incubated with an excess of 11-*cis*-retinal for 4 h to reconstitute the pigment. The pigment was extracted with 1% dodecylmaltoside (DM) in 50 mM HEPES buffer (pH 6.5) containing 140 mM NaCl (buffer A), bound to 1D4-agarose, washed with 0.02% DM in buffer A (buffer B), and eluted with buffer B containing the C-terminal peptide of bovine rhodopsin. Pigment-containing HEK293 membrane were prepared as described elsewhere^44^. Briefly, HEK293S cells were collected two days after transfection and the pigment of amphioxus Go-rhodopsin was reconstituted with an excess of all-*trans*-retinal overnight. Pigment-containing cell membranes were collected by sucrose flotation with buffer A containing 45% sucrose.

### Spectroscopic measurements

Absorption spectra of purified samples were measured with a spectrophotometer (UV2450; Shimadzu, Japan). A 1-kW halogen lamp (Philips) was used for illumination of samples in combination with interference filters (400, 420, 550 nm; Toshiba), cut-off filters (R-62, O-56, O-53; AGC TECHNO GLASS), or a UV glass filter (UTVAF-50S-36U; Sigma Koki, Japan). Alteration of pH was performed by adding CAPS-NaOH buffer and Na_2_HPO_4_ for alkaline conditions and NaH_2_PO_4_ and HCl for acidic conditions. pH values of samples were measured with a pH meter (B-211; HORIBA, Japan). Difference spectra of pigments in the membrane preparation were measured using a spectrophotometer (V-750 UV-VIS Spectrophotometer; JASCO International, Japan) equipped with an integrating sphere. A 100-W halogen lamp was used for illumination of samples in combination with a cut-off filter (O-59, AGC TECHNO GLASS). All measurements were carried out at 4 °C.

### HPLC analysis and calculation of absorption spectra

HPLC analysis was performed as described elsewhere^45^. Briefly, retinal chromophore in a sample was converted into retinal oxime with hydroxylamine and then extracted with *n*-hexane^32^. The samples were injected into a silica column (YMC-Pack SIL, particle size 3 μm, 150 × 6.0 mm) and eluted with n-hexane containing 15% (v/v) ethyl acetate and 0.15% (v/v) ethanol. Concentration of each isomers of retinal oximes was determined with the molecular extinction coefficient values (*syn*-11-*cis*, 35,000 M^−1^ cm^−1^; *syn*-all-*trans*, 54,900 M^−1^ cm^−1^; *anti*−11-*cis*, 29,600 M^−1^ cm^−1^; *anti*-all-*trans*, 51,600 M^−1^ cm^−1^). Spectra of photoproducts were calculated from spectra of samples before and after irradiation and their retinal composition. A difference spectrum (Δ*A*) and the ratio of retinal that photo-converted from 11-*cis* to all-*trans* forms (Δ*R*) were calculated with absorption spectra of the dark state (*A*_d_) and irradiated sample (*A*_i_) and their retinal composition as follows:

$$\Delta A = A_{\mathrm{i}}-A_{\mathrm{d}},$$

$$\Delta R = \left( {R_{\mathrm{i}}^{{\mathrm{at}}}-R_{\mathrm{d}}^{{\mathrm{at}}}} \right){\mathrm{/}}R_{\mathrm{d}}^{11{\mathrm{c}}},$$

where *R*_i_^at^, *R*_d_^at^, and *R*_d_^11c^ represent for ratios of all-*trans* form in irradiated (*R*_i_^at^) and dark (*R*_d_^at^) samples and 11-*cis* form in the dark sample (*R*_d_^11*c*^), respectively. With these values, we calculated an absorption spectrum of the photoproduct (*A*_p_), in which all the original 11-*cis* form would convert to all-*trans* form, with the following equation:

$$A_{\mathrm{p}} = A_{\mathrm{d}} + \Delta A{\mathrm{/}}\Delta R.$$

### Calcium imaging assay

Calcium imaging assay was performed as described elsewhere^46^. In brief, COS-1 cells (CRL-1650, ATCC) were transfected with a plasmid DNA by using FuGENE HD Transfection Reagent (Promega). After overnight incubation at 37 °C, the transfected cells were incubated in Krebs–Ringer HEPES buffer containing 5 µM Fura 2-AM (Dojindo, Japan) and 1 µM 11-*cis*-retinal for 1 h and then rinsed. Fura-2 fluorescence was measured using a fluorescence microscope (BX-51, Olympus) with a CMOS camera (ORCA-Flash4.0, HAMAMATSU) and MetaMorph software (Molecular Devices).

### Homology modeling of 3D structure

A 3D model of spider Rh1 was built by homology modeling using the crystal structure of squid rhodopsin (PDBid: 2Z73^19^) as a template. First, the sequences of spider Rh1 (UniProt id: B1B1U5) and squid rhodopsin (Uniprot id: P31356) (~35% sequence identity) were aligned using Clustal Omega^47^. This initial alignment was manually refined using Chimera^48^ to adjust some of the gaps in the loop regions. Using this alignment and the squid rhodopsin template, a 3D model of spider Rh1 was built using using Modeller v9.20^49^. The putative cysteine bridge between Cys123 in transmembrane helix III and Cys200 in the second extracellular loop was explicitly defined during model building. All models were subjected to 300 iterations of variable target function method optimization and thorough molecular dynamics and simulated annealing optimization and scored using the discrete optimized protein energy potential. The 20 best-scoring models were analyzed visually, and a suitable model (in term of low score and structure of the loops) was selected. Finally, 11-*cis*-retinal was modeled in the binding site using molecular graphics software (The PyMOL Molecular Graphics System, Version 2.0 Schrödinger, LLC) and the structure of squid rhodopsin as a template. Figures displaying three-dimensional structures were prepared with PyMOL.

### Reporting summary

Further information on research design is available in the Nature Research Reporting Summary linked to this article.

## Supplementary information

Supplementary Information

Reporting Summary

## Data Availability

The data that support the findings of this study are available from the corresponding author on reasonable request.
