# Supplementary material for: The counterion–retinylidene Schiff base interaction of an invertebrate rhodopsin rearranges upon light activation
Source: Commun Biol. 2019 May 13;2:180. doi: 10.1038/s42003-019-0409-3 (PMC6513861; doi:10.1038/s42003-019-0409-3)
Supplement: Supplementary file 1 — Supplementary Information [file 42003_2019_409_MOESM1_ESM.pdf]

Supplementary Figures

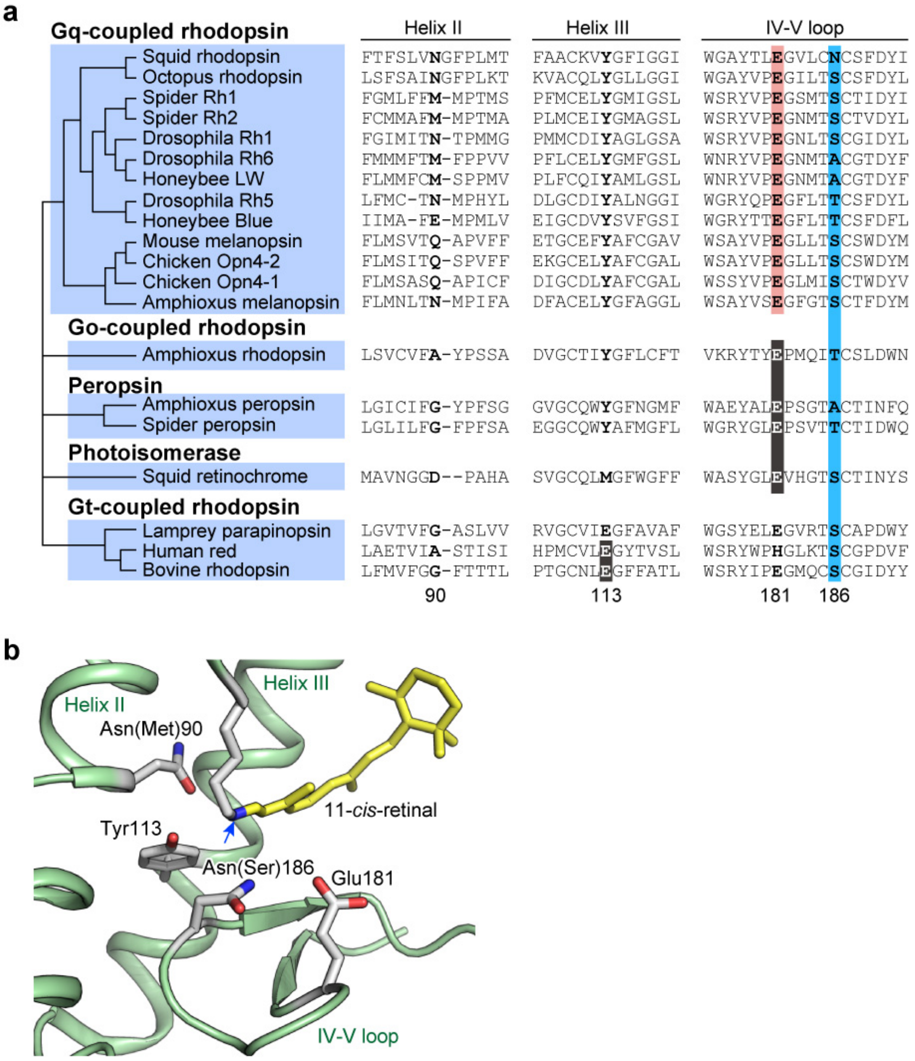

Supplementary Fig. 1 (**a**) Phylogenetic relationship and multiple alignment of amino acid sequences of opsins from several subfamilies. In the alignment, only amino acids around positions 90, 113, 181, and 186 (in transmembrane helices II and III, and in the IV-V loop) are shown. In Go- and Gt-coupled rhodopsin, peropsin, and photoisomerase, Glu113 or Glu181 (highlighted in grey) serve as the counterion for the PSB. Glu181 is highly conserved in Gq-coupled group (highlighted in orange). Ser/Thr at position 186 (highlighted in blue) is conserved among many opsins. (**b**) Crystal structure of squid rhodopsin (Protein Data Bank identifier: 2Z73) around the retinylidene SB (arrow). The side chains of amino acid residues within 5 Å of the SB and the putative counterion Glu181 are shown as sticks. The amino acid names in parentheses denote the residues at these positions in spider Rh1.

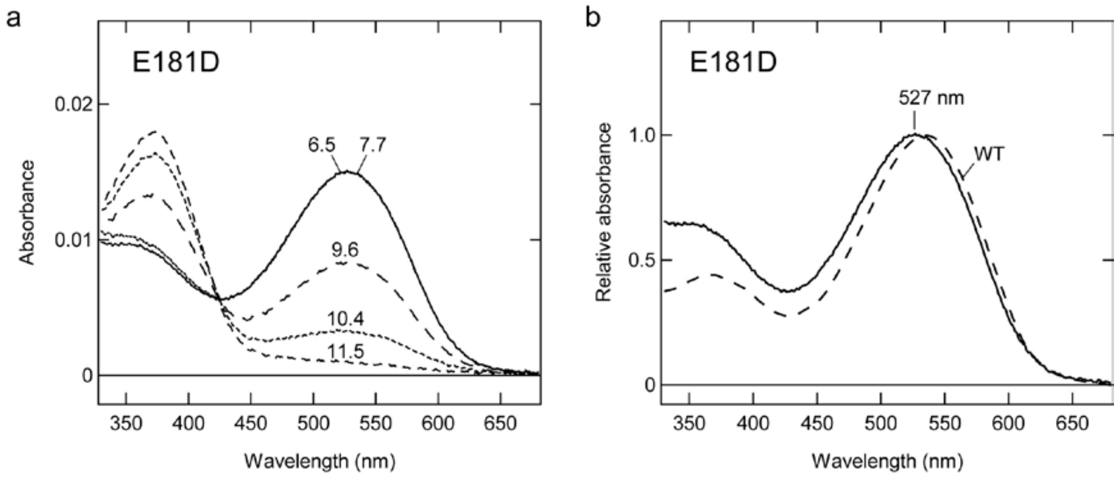

Supplementary Fig. 2 Absorption spectra of the E181D mutant of spider Rh1 in the dark state. (**a**) Absorption spectra at different pH conditions. (**b**) Comparison of absorption spectra of the dark state between the E181D mutant and wild type.

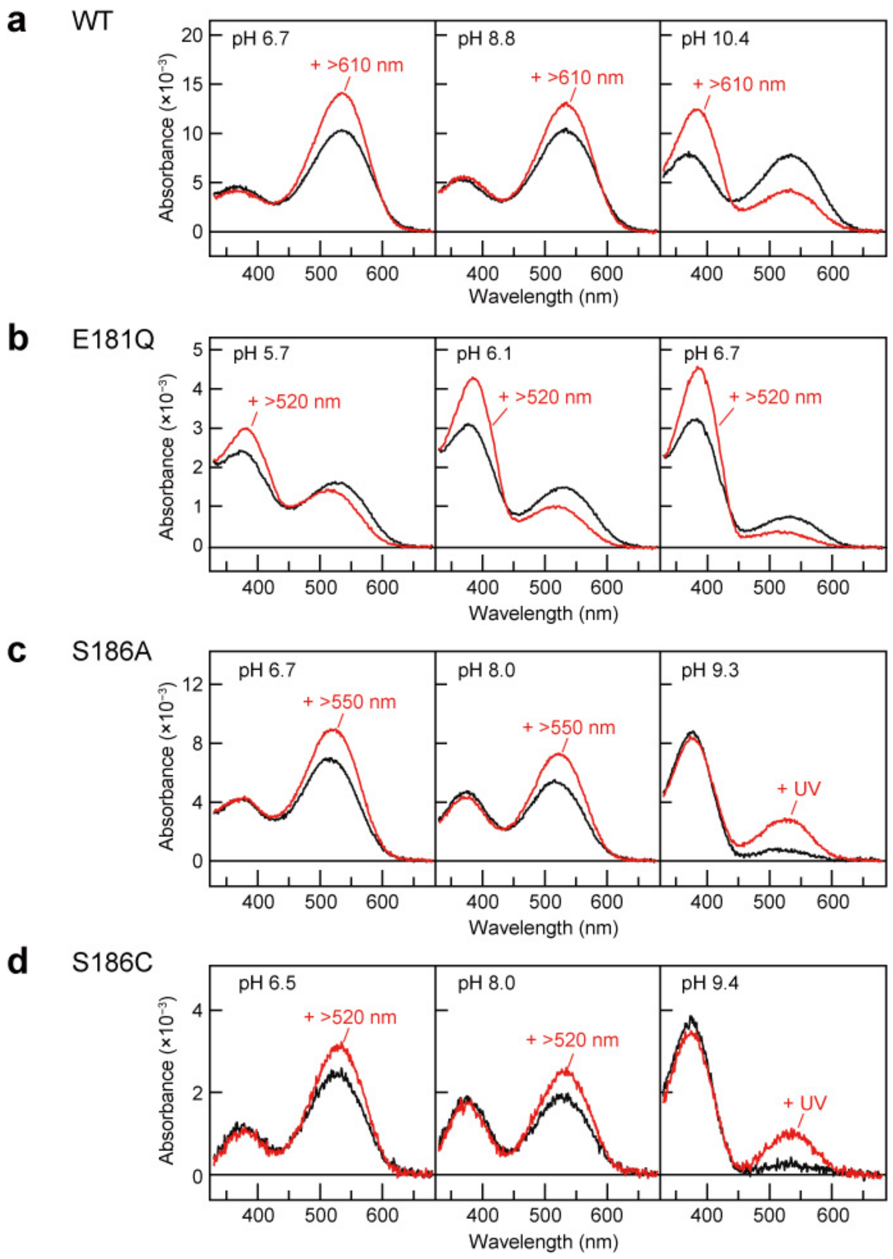

Supplementary Fig. 3 Absorption spectra of WT (**a**) and the E181Q (**b**), S186A (**c**), and S186C (**d**) mutants before (black) and after (red) illumination at different pH values. In the wild type, the decrease in absorbance in the visible region at pH 10.4 shows that the SB pKa in the photoproduct is lower than in the dark state. In contrast, the spectra of the S186A and S186C mutants at around pH 9 demonstrate that their SB pKa values are higher in the photoproducts.

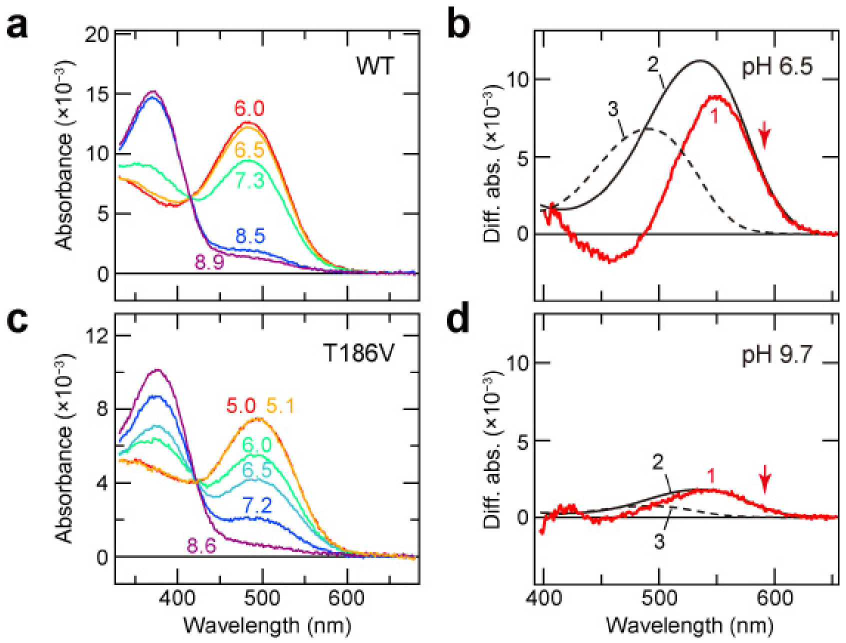

Supplementary Fig. 4 Spectroscopic analyses of amphioxus Go-rhodopsin in the dark state and the photoproduct at different pH values. (**a**, **b**) Absorption spectra of purified pigments of WT (**a**) and T186V mutant (**b**). The pH value for each measurement is indicated next to the curve. (**c**, **d**) Spectroscopic measurements of the photoproduct reconstituted with all-*trans*-retinal in the membrane preparation. In these conditions, amphioxus Go-rhodopsin forms a pigment bearing all-*trans*-retinal that is identical to the red-shifted photoproduct produced by light absorption[1]. Illumination with orange light (>580 nm) at a neutral pH converts almost all of the photoproduct to the dark state[2], causing a positive peak around 550 nm in different spectra. Curve 1 shows the difference spectra (reflecting conversion from the initial photoproduct (curve 2, schematic spectrum) to the dark state (curve 3, schematic spectrum)) of WT between before and after illumination with orange light (>580 nm) at pH 6.5 (**c**) and 9.7 (**d**). Arrows mark the wavelength of 590 nm, where difference spectra are only derived from the photoproduct. We measured such different spectra of WT and T186V mutant at different pH values and plotted the relative values of absorbance at 590 nm (Fig. 5**b**).

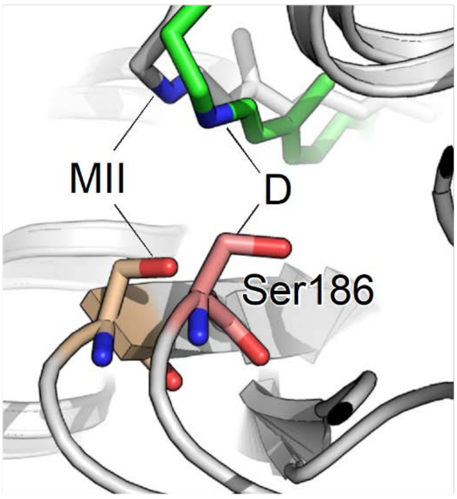

Supplementary Fig. 5 Relationship of Ser186 and the PSB in the dark and Meta II states of bovine rhodopsin. Superposition of the dark (D; PDBid: 1GZM[3]) and the Meta II (MII; PDBid: 4A4M[4]) states is shown. The distances between Ser186 and the PSB are 3.4 Å in the dark state and 5.0 Å in Meta II state.

Supplementary Table

Estimated absorption maxima of spider Rh1 and its mutants at pH 6.5.

	λmax[1] of the dark state (nm)	λmax of the photoproduct (nm)
WT	535	535
E181Q	525[2]	≈500[3]
Y113F	532	n.d.[4]
S186A	515	526
S186C	524	533
S186T	528	n.d.
S186N	515	n.d.
S186G	525	n.d.
S186F	380	540

[1]in the visible region
[2]determined at pH 5.4
[3]determined at pH 5.7
[4]n.d., not determined.

References

1. Tsukamoto H., Terakita A., Shichida Y. A rhodopsin exhibiting binding ability to agonist all-trans-retinal. *Proc Natl Acad Sci USA* **102**, 6303-6308 (2005).

2. Terakita A., Koyanagi M., Tsukamoto H., Yamashita T., Miyata T., Shichida Y. Counterion displacement in the molecular evolution of the rhodopsin family. *Nat Struct Mol Biol* **11**, 284-289 (2004).

3. Li J., Edwards P. C., Burghammer M., Villa C., Schertler G. F. Structure of bovine rhodopsin in a trigonal crystal form. *J Mol Biol* **343**, 1409-1438 (2004).

4. Deupi X., *et al.* Stabilized G protein binding site in the structure of constitutively active metarhodopsin-II. *Proc Natl Acad Sci USA* **109**, 119-124 (2012).
